# Epigenetics, c-Myc and Aggressive B-cell Lymphomas

**DOI:** 10.18632/oncotarget.748

**Published:** 2012-11-13

**Authors:** Yizhuo Zhang, Xiaohong Zhao, Jianguo Tao

**Affiliations:** Department of Hematology, Tianjin Cancer Institute and Hospital, Tianjin Medical University, Tianjin, China; Department of Hematopathology and Laboratory Medicine, and Experimental Therapeutics Program, H. Lee Moffitt Cancer Center and Research Institute and the University of South Florida, Tampa, Florida; Department of Hematopathology and Laboratory Medicine, and Experimental Therapeutics Program, H. Lee Moffitt Cancer Center and Research Institute and the University of South Florida, Tampa, Florida

c-Myc (hereafter referred to as Myc) protein plays a fundamental role in cell cycle regulation, proliferation, differentiation, and apoptosis by modulating the expression of a large number of targets [1]. Expression of Myc is frequently deregulated in human cancers and is often associated with aggressive disease. In non-Hodgkin lymphomas, although Myc has been described as a defining feature and the driving oncogene for Burkitt lymphoma, Myc overexpression has also been recognized in other aggressive B-cell lymphomas and has been linked to adverse prognosis [2]. In addition to Burkitt lymphoma, these aggressive lymphomas include Myc-associated diffuse large B-cell lymphoma (DLBCL), double-hit lymphoma, acute lymphoblastic lymphoma, a blastic variant of mantle cell lymphoma (MCL), transformed follicular lymphoma, and plasmablastic lymphoma. However, mechanisms underlying the sustained activation of Myc and subsequent contribution to clinical fatality are unclear. Recently, Myc transcriptional network has been shown to also include microRNAs [3]. These small non-protein-coding, single-stranded RNAs function as negative regulators of mRNA and affect virtually every aspect of tumorigenesis [3]. Although Myc can alter a large set of miRNAs, rather than activation, the majority of identified miRNAs are repressed by Myc [4]. Among these repressed miRNAs, many are putative tumor suppressors, such as let-7, miR-15a/16-1, miR-26a, miR-29, and miR-34a. Each of these miRNAs has been associated with anti-proliferative, pro-apoptotic, and/or anti-tumorigenic activity. Reactivating these miRNAs in Myc-transformed B lymphoma cell lines dramatically inhibits tumorigenesis [4], indicating that reconstituting lymphoma with these tumor suppressor miRNAs could be therapeutically beneficial in Myc-associated lymphomas. Therefore, it is likely that Myc hyperactivity contributes to widespread repression of miRNA expression and that Myc-driven miRNA repression underlies the molecular mechanisms related to lymphoma aggressive transformation.

**Figure 1:**
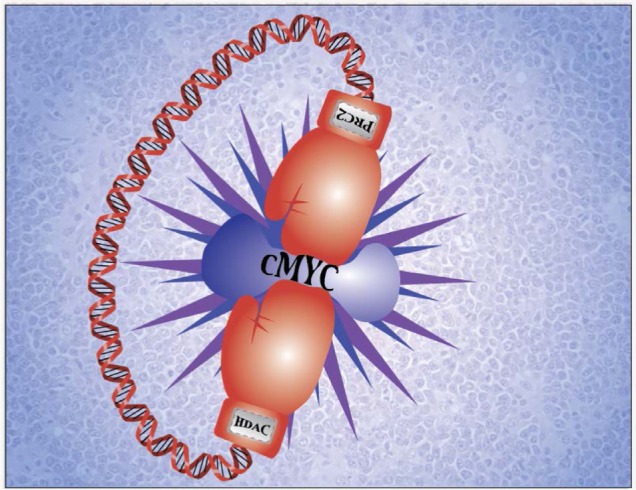
Epigenetically targeting c-Myc and c-Myc-regulated genes for aggressive B-cell lymphoma therapy c-Myc plays a central role in aggressive B-cell lymphoma pathogenesis. c-Myc and c-Myc target miRNAs are regulated through histone modifications and can be epigenetically targeted by HDAC and PRC2 inhibitors for aggressive B-cell lymphoma therapy.

We recently explored the role of epigenetic regulation in Myc-mediated miRNA repression [5]. We revealed 1) loss or low expression of c-Myc-regulated miRNAs in aggressive B-cell lymphomas; 2) reverse correlation of tumor suppressor miRNAs such as miR-26a and miR29-a-c with Myc as well as cell proliferation, CDK6, and IGF-1R expression; 3) ectopic expression of miR-26a, miR-29 suppression of CDK-6, and IGF-1R and Myc-driven cell proliferation in aggressive lymphoma cell lines and primary lymphomas; and 4) miR-26a and miR-29 repression as a result of Myc/HDAC3 and EZH2 (a catalytic component of polycomb repressive complex 2, PRC2) interaction. Results of these studies have led to the identification of a novel model for interplay between Myc, HDAC3, PRC2, and miRNAs and their contribution to Myc-associated lymphomagenesis, and HDAC3/EZH2/miRNAs as novel therapeutic targets. Myc, HDCA3, and PRC2 form a repressive complex tethered to miR-29a/b1 and miR-29b2/c promoter regulatory elements to epigenetically repress transcription of these miRNAs in Myc-expressing lymphoma cells and that subsequent miR-29 down-regulation results in induction of oncogenic proteins (CDK6 and IGF-1R) and Myc-driven lymphomagenesis. Furthermore, we demonstrated that Myc contributes to the upregulation of EZH2 via repressing EZH2-targeting miR-26a and that EZH2 in turn induces Myc expression via Myc-targeting miR-494, thereby generating *a positive feedback loop* to ensure persistent high protein levels of Myc and EZH2 and further repression of miR-29, which could be involved in maintaining sustained Myc activation and malignant phenotype. More importantly, these findings provided therapeutic rational and opportunities for treating aggressive B-cell lymphomas by attacking Myc through epigenetically modulating Myc upstream “targeting-Myc miRNAs” such as miR-494 as well as attacking Myc downstream “Myc-targeting miRNAs” such as miR-29 through histone modification (Figure 1). Indeed, our study showed that treatment with the pan-HDAC inhibitor SAHA, the EZH2 inhibitor DZNep, and their specific siRNAs disrupted Myc hyperactivity, resulting in enhanced restoration of miR-29a-c expression, down-regulation of miR-29 targeting genes CDK6 and IGF-1R, and suppression of lymphoma cell growth ex vivo and in vivo. In line with our findings, it was recently shown that the EZH2 inhibitor GSK126 effectively inhibited the proliferation of EZH2-mutant DLBCL cell lines and markedly inhibited the growth of EZH2-mutant DLBCL mouse xenografts, supporting EZH2 inhibition as a therapeutic strategy for lymphoma therapy [6].

Different approaches have been taken to find ways to therapeutically target Myc in cancer, and however, efforts to target Myc activity have proven unsuccessful. Until recently, Bradner and colleagues found a way to inhibit *Myc* indirectly [7]. They devised JQ1, a small molecule that prevents bromodomain from binding to acetylated histone, silencing c-Myc transcription. JQ1 functions as a specific inhibitor of bromodomain-containing protein 4 through an interference of acetyl-lysine recognition domains (bromodomains). When applied to Myc harboring multiple myeloma and Burkitt lymphoma, they showed that treatment with JQ1 leads to a profound cell-cycle arrest and apoptosis of the cell lines with associated reduction in *Myc* transcription and protein expression ex vivo and in murine models of multiple myeloma [7]. Moreover, Mertz and colleagues extended these findings to reveal a survival benefit in murine xenograft models of Burkitt lymphoma [8]. However, silencing Myc with JQ1 for combinatorial therapy of aggressive B-cell lymphomas has not been reported. A study by us strongly supported the further development and testing of a combination of JQ1 with EZH2 and/or HDAC3 inhibitor against aggressive B-cell lymphomas [5].

Most recently, seminal studies have demonstrated that PI3K is indispensable for lymphoma survival and that it cooperates with c-Myc in Burkitt lymphomagenesis [9-10]. These important findings support that B-cell receptor (BCR)-PI3K axis and c-Myc pathway act in concert to contribute to lymphoma treatment resistance and aggressive progression. With newly established BCR signaling inhibitors, it is inspiring to exploit synthetically lethal targeting of BCR-PI3K axis (using PCI-32765, CAL101), and Myc pathway (JQ-1, DZNep) for these lymphomas. Functional identification of Myc activation mechanisms and its interplay with other survival pathways such as BCR will allow us to gain insight into lymphoma survival and progression and provide novel biological targets for aggressive lymphomas.
